# The outcome of postoperative radiation therapy following plastic surgical resection of recurrent ear keloid: a single institution experience

**DOI:** 10.1186/s43046-022-00105-8

**Published:** 2022-01-24

**Authors:** Reham Mohamed, Abosaleh Abosaleh Elawadi, Reham Al-Gendi, Safa Al-Mohsen, Shabeer Wani, Ahmed Wafa

**Affiliations:** 1 Radiation Oncology Department, National Cancer Institute, Cairo University, Cairo, Egypt; 2 Radiation Oncology Department, Comprehensive Cancer Center, King Fahad Medical City, Riyadh, Saudi Arabia; 3 Medical Physics Section, Radiation Oncology Department, Comprehensive Cancer Center, KFMC, Riyadh, Saudi Arabia; 4 Medical Physics Section, Clinical oncology and Nuclear Medicine Department, Faculty of Medicine, Mansoura University, Mansoura, Egypt; 5 Plastic Surgery Department, King Fahad Medical City, Riyadh, Saudi Arabia

**Keywords:** Keloid, Postoperative, Radiotherapy, Orthovoltage

## Abstract

**Background:**

Ear keloids are abnormal continuously growing healing process following cutaneous injury. Surgical excision is the standard treatment strategy; however, 50–80% of cases develop recurrence. Adjuvant radiotherapy (RT) is commonly offered with a marked decrease in the recurrence rate. The variation in RT protocols used in different studies leads to a bias of results analysis. The aim is to present our experience of using surgical excision with postoperative radiotherapy for recurrent ear keloids. Also, studying different variables especially dose and keloid size that affects recurrence rate. Radiotherapy complications were reported and assessed.

**Patients and methods:**

Keloids between 2006 and 2021 were retrospectively reviewed. Fifty-five ear keloids out of 83 cases who received RT after surgical excision were included in the study. Different dose regimens including 13 Gy/1fx, 8 Gy/1fx, 10 Gy/2fx, 15 Gy/3fx, and other fractionated regimens were used. The Median follow-up period was 35 months. Recurrence-free rate (RFR), side effects, and prognostic factors were assessed.

**Results:**

The overall 2-year RFR was 88 ± 5%. The 2-year RFR was 83 ± 8% for dose regimens with biological effective dose (BED) ≤ 40 and 92 ± 5% for regimens with BED > 40 Gy with an insignificant *p* value. The 2-year RFR was 74 ± 10% compared to 97 ± 3% for keloids > 2 cm and keloids ≤ 2 cm respectively (*p* value 0.02). The higher dose used for keloids with > 2 cm size significantly improved RFR. The orthovoltage therapy showed marginally better 2-year RFR compared to electron beam therapy; however, statistically insignificant (*p* value 0.09). The side effects were minimal with no reported second malignancy or serious G3-4 complications.

**Conclusion:**

Excision followed by RT is a safe and effective treatment for recurrent ear keloids. Low and modest radiation doses are effective; however, a higher dose is recommended for keloids > 2 cm. We recommend a prospective larger-scale study to test the effect of dose and keloid size on the treatment results.

## Background

Radiotherapy for benign diseases started shortly after the x-ray discovery in 1896. Keloid is one of such diseases that showed decreased recurrence rate by adding postoperative radiotherapy (PORT) [1, 2]. The continuous abnormal healing that mostly exceeds the boundary of the initial wound edges characterizes post-traumatic keloid formation [3]. The ear is considered the commonest site affected by keloid scarring with cosmetic complaints and infrequent pain and pruritis [4, 5]. The proposed treatment approaches included non-invasive and invasive strategies, such as compression, intralesional injections of corticosteroids, intralesional injection of pharmaceutical agents like verapamil and bleomycin, topical therapy, laser treatment, intra-lesional cryotherapy, and surgical excision. However, it is an invasive option, resection remains the standard approach for recurrent cases following the failure of conservative measures [6–12].

Unfortunately, many studies showed that the incidence of postoperative local recurrence ranges from 50 to 80%. Moreover, multiple surgical resections led to bigger recurrences in most of the clinical scenarios [13–15]. Adjuvant PORT aiming to prevent local recurrence showed its effectiveness and superiority over other options [16–18]. Radiation targets immature fibroblasts which are relatively radiosensitive compared to normal fibroblasts leading to suppression of fibroblast proliferation and hence inhibition of collagen synthesis [15, 16]. Accordingly, radiation therapy (RT) should be considered as early as possible within 3 days following resection before fibroblast maturation [1, 13, 16]. This standard clinical practice of surgery followed by early PORT dated and proposed since 1981 by Ollestein et al. [19]. The proposed radiation dose varies in the literature with no consensus ranging from 7 Gray (Gy) to 13 Gy as a single dose or even fractionated ranging from 10 to 20 Gy. Most studies kept the high dose per fraction (fx) as a general concept regardless of the way of fractionation being keloids have low mitotic index [20–27]. Despite its rarity, radiation-induced second malignancy is a potentially serious side effect in such benign diseases that warrants RT optimization and discussion with the patients upon offering PORT [22, 23].

### Aim of the study

The study aims to present our experience of using surgical excision with PORT for the treatment of recurrent ear keloids. The variables that possibly affect treatment outcomes were studied. The possible radiation-induced side effects and complications were evaluated.

### Patients and methods

**Fig. 1:**
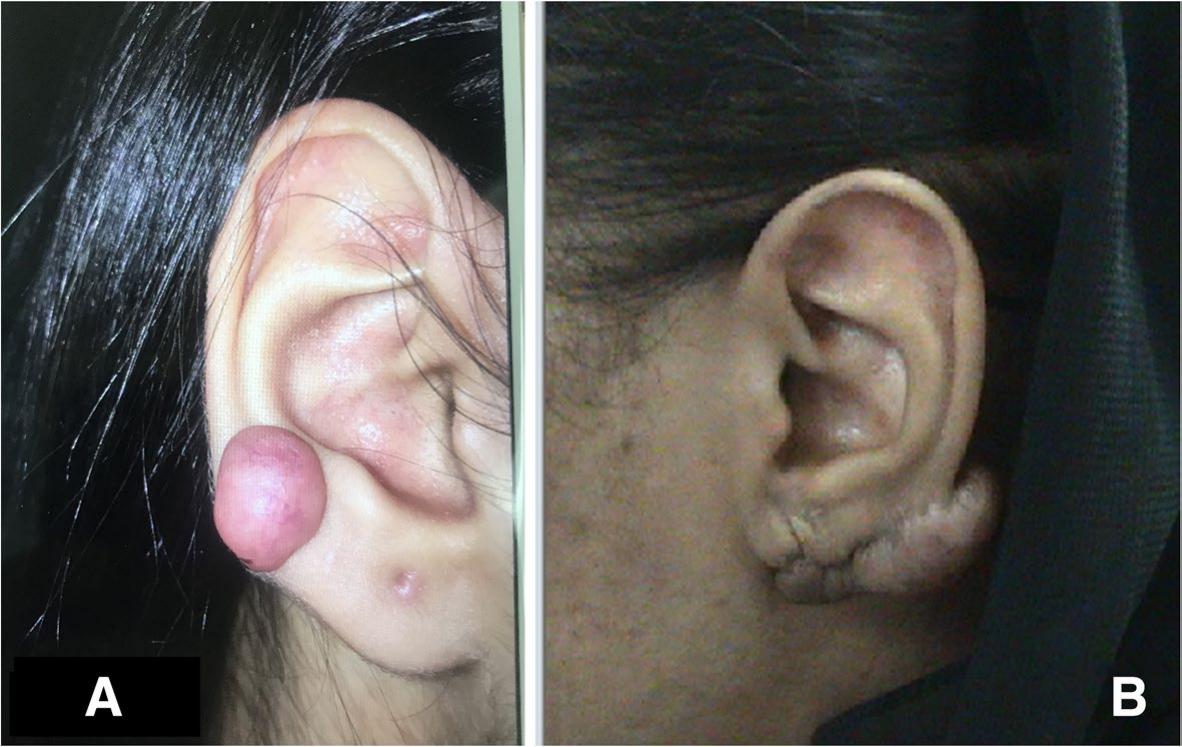
Patients with recurrent ear keloids of different sizes. **A** Showed patient with RT ear keloid less than 2 cm. **B** Showed patient with left ear keloid more than 2 cm

The patients presented by recurrent ear keloids (Fig. 1) and treated by surgical resection and PORT from 2006 till 2021 at our hospital were retrospectively reviewed. Institutional Review Board (IRB) approval was obtained before data collection. The medical records and radiation therapy files were used to collect the following information; disease laterality, radiation dose, number of fractions, dose per fraction, radiation energy, the interval between surgery and radiotherapy, local recurrence, early and late radiation-induced side effects. Our data were compared with other data published in the literature.

#### Surgical details

Excision of a keloid may stimulate additional collagen synthesis, prompting quick recurrence as a possible larger keloid than the initial one. So, the strategy of limiting tissue handling is followed. All adult patients underwent surgery under local anesthesia. The common practice at our institution is complete extramarginal excision leaving 5-mm margins of healthy skin as recommended worldwide. The incisions and wound edges are planned to be parallel to the main folding lines of the skin to decrease the recurrence rates. After undermining the surrounding skin for easy closure, the wound edges were closed under tension with absorbable subdermal and nonabsorbable subcuticular sutures.

#### Radiotherapy details

The radiation treatment was delivered at our department by using either electron beam therapy or orthovoltage x-ray beam. The linear accelerator is a dual-energy HDX machine (Varian Medical System, Palo Alto, USA). The orthovoltage machine is Xstrahl 300, SN Gm0372. This machine produces 9 clinical energies of x-ray beam from 60, 80, 100, 120, 150, 180, 200, 250 and 300 kilovoltage peak (kVp) with filters F1, F2, F3, F4, F5, F6, F7, F8, and F9 respectively. Patients were treated using open or closed applicators at focal spot distance 30 cm or 50 cm respectively. Open circular applicators are used with energies 60, 80, and 100 kilovolt (kV) while closed square or rectangular applicators are used with the remaining energies of more than 100 kV.

**Fig. 2:**
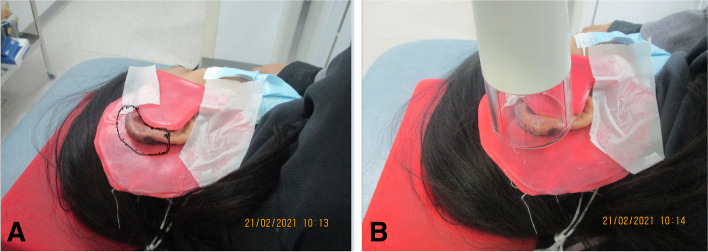
Patient with Rt ear keloid treated by orthovoltage. **A** Showed patient with RT ear keloid with target volume determined and waxed lead shield used for normal tissue protection. **B** Showed the same patient with orthovoltage applicator prior to treatment

The patients were treated in the lateral position or supine position with the head turned to the other side so that the affected ear is facing up. A suitable head-rest device is used to allow proper comfortable reproducible positioning. The target volume was determined clinically including the scar plus a 1 to 1.5 cm margin (Fig. 2A). The depth was chosen clinically and mostly around 0.5–1.0 cm. Waxed lead cutout shields were positioned around the delineated target volume to block the normal tissue. Also, a waxed lead shield is placed behind the ear to protect the neck and brain and to avoid backscatter radiation (Fig. 2B). The gantry may be rotated so that the beam exits away from the inner and middle ear if applicable. In the case of treatment by 6 mega-electron volt (MeV) electron beam, a bolus of 0.5 cm thickness was applied to keep skin dose close to 100%. The dose was prescribed to 85–90% isodose line. In case of treatment with higher energies of electron beam, the skin dose was calculated, and mostly bolus is not applied. In case of orthovoltage treatment, the proper energy (filter) was used as per treatment depth with the dose prescribed to 90–95% isodose line. Different fractionation schedules were used as per the treating physician.

For the sake of comparison, we used the equivalent dose in 2-Gy fractions (EQD2) of 20 Gy with biological effective dose (BED) 40 to stratify the regimens used in our department. As a benign disease, we considered the α/β ratio for ear keloid to be 2.08 similar to late reacting tissues [28]. The regimens with EQD2 ≤ 20 Gy (BED ≤ 40) included 8 Gy/1fx and 10 Gy/2fx compared to regimens with EQD2 > 20 Gy ((BED > 40) that included 13 Gy/1fx, 15 Gy/3fx, 16 Gy/4fx, and 18 Gy/3fx.

The patients were followed up with radiation or plastic surgery departments every 3–6 months. We used telephone interviews for some patients who could not attend regular follow-up visits. Recurrence is defined as a reappearance of the keloid or progression of the residual scar elevation [16]. The recurrence-free duration is measured from the date of surgical excision till the date of local recurrence. The radiation-induced skin reactions were evaluated using Radiation Therapy Oncology Group (RTOG) grading scale [24].

#### Statistical analysis

Statistical package for social science version 21 (SPSS v21) was used for statistical analysis and the Kaplan-Meier method was used to estimate recurrence-free rate. The log-rank test was used to compare recurrence rates between groups. *P* values of < 0.05 were considered statistically significant. All different variables were studied and correlated with local recurrence. The student’s *t* test was used for the analysis of continuous variables. The chi-squared test and Fisher’s exact test for discrete variables were used to compare proportions.

## Results

Eighty-three keloid cases registered at our radiation oncology department were screened. The cases with keloids outside the ear and hypertrophic scars were excluded and a total of 55 recurrent ear keloids were included in the study with the following characteristics shown in Table 1. Majority of patients were females (91%) with a mean age of 24 ± 7 years and a range from 17 to 66 years. The main presentation was painless mass in 89% of cases. Thirty-eight (69%) cases had a history of 2 to 3 resections before re-excision and PORT (Table 1).

**Table 1:** Patients’ characteristics

Variable (total no of cases; *n* = 55)	*n* (%)
Age	
Mean age ± SD (range) in years	24 ± 7 (17–66)
Sex	
Female	50 (91%)
Male	5 (9%)
Presentation	
Mass	49 (89%)
Mass and pain	6 (11%)
No of resections prior to definitive treatment	
1 resection	10 (18%)
2 resections	21 (38%)
3 resections	17 (31%)
> 3 resections	7 (13%)
Duration between initial presentation and RT	
Mean duration ± SD (range) in months	71 ± 43 (19–180)
Referral hospital	
Internal referral	33 (60%)
External referral	22 (40%)
Involved ear	
Rt ear	32 (58%)
Lt ear	23 (42%)
Current clinical status	
Free	49 (89%)
Recurrent	6 (11%)
Treatment used before RT	
Surgery alone	7 (13%)
Surgery and steroid injection	48 (87%)
Size	
0.0–1.0 cm	4 (7.3%)
> 1.0–1.5 cm	20 (36.4%)
> 1.5–2.0 cm	9 (16.3%)
> 2.0–4.0 cm	17 (31%)
> 4.0 cm	5 (9%)

The mean duration between initial presentation and PORT was 71 ± 43 months ranging from 19 to 180 months. The keloid size before the last surgical resection was > 2 cm in 40% of cases. Forty cases (72.8%) received a single radiation dose either 8 Gy or 13 Gy. Two cases received 10 Gy/2fxs, 8 cases received 15 Gy/3fxs, 4 cases received 16 Gy/4fxs, and one patient received 18 Gy/3fxs. Forty-one cases out of the 55 cases received orthovoltage while 25% of the cases received electron beam therapy. The energy of 100 kV with 3.2 mm aluminum half-value layer (HVL) was used for treating 64% of the cases. All of our patients received radiation within 24 h of surgery. The details of radiation delivered are illustrated in Table 2.

**Table 2:** Radiation details

Variable (total no of cases; *n* = 55)	*n* (%)
RT prescription dose	
8 Gy single dose	20 (36.4%)
13 Gy single dose	20 (36.4%)
10–18 Gy fractionated doses	15 (27.2%)
Dose per fractions	
8 Gy	20 (36.4%)
13 Gy	20 (36.4%)
4 Gy	4 (7.3%)
5 Gy	10 (18%)
6 Gy	1 (1.9%)
Type of RT	
Orthovoltage	41 (75%)
Electron beam	14 (25%)
Orthovoltage applicator size (41 patients)	
3 cm	5 (12%)
4 cm	20 (49%)
5 cm and more	16 (39%)
Energy	
100 kV	35 (64%)
150 kV	4 (7.3%)
180 kV	2 (3.6%)
6 MeV	12 (21.5%)
9 MeV	2 (3.6%)
Acute side effects	
G1	37 (67%)
G2	5 (9%)
Not assessed	13 (23%)
Late side effects	
No late reactions	43 (78.1%)
G1	8 (14.6%)
Not assessed	4 (7.3%)

**Fig. 3:**
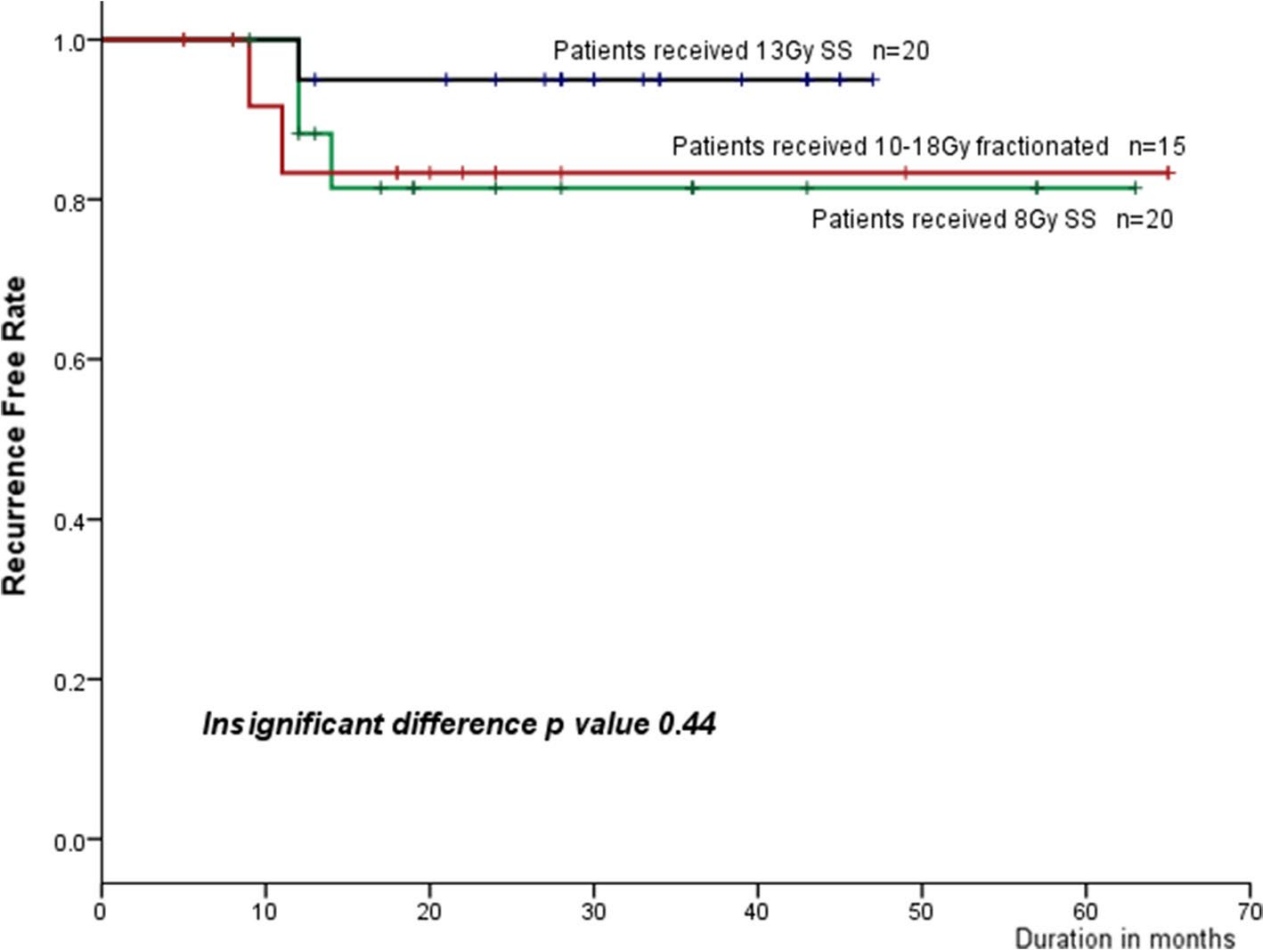
The effect of different dose regimens on the recurrence rate. The curve showed insignificant difference between the used dose regimens; 13 Gy/1fx, 8 Gy/1fx, and 10–18 G fractionated regimens

The mean follow-up period was 35 ± 16 months ranging from 8 to 72 months. At the time of assessment, 49 cases were free of local recurrence with 2-year recurrence-free rate (2y-RFR) 88 ± 5%. The different dose regimens used did not affect the RFR significantly with a *p* value of 0.44 as illustrated in Table 3 and Fig. 3.

**Table 3:** Recurrence free rateVariable (total no of cases; *n*=55)

	2y-RFR ± SD	*P* value
All patients	88 ± 5%	
Group received 8Gy SS^a^	81 ± 10%	
Group received 13Gy SS^a^	95 ± 5%	
Group received 10-18Gy Fractionated	83 ± 11%	0.44
EQD2		
≤ 20 Gy	83 ± 8%	
> 20 Gy	92 ± 5%	0.37
Size		
Keloid ≤ 2 cm	97 ± 3%	
Keloid > 2 cm	74 ± 10%	0.02
Type of radiation		
Orthovoltage	92 ± 4%	
Electron beam	72 ± 14%	0.09
Size and dose effect		
Keloid ≤ 2 cm		
Group received 8 Gy SS	91 ± 9%	
Group received 13 Gy SS	100 ± 0%	0.3
Keloid > 2 cm		
Group received 8 Gy/1fx	56 ± 24%	
Group received 13 Gy/1fx	88 ± 12%	0.05
Mean follow up period in months (range) (8–72)	35 ± 16	
Mean time to recurrence in months (range) (5–65)	26 ± 14	

^a^SS = single shot. Comparing patients of 8Gy SS and patients of 13Gy SS separately is insignificant also with *p* value 0.2

**Fig 4:**
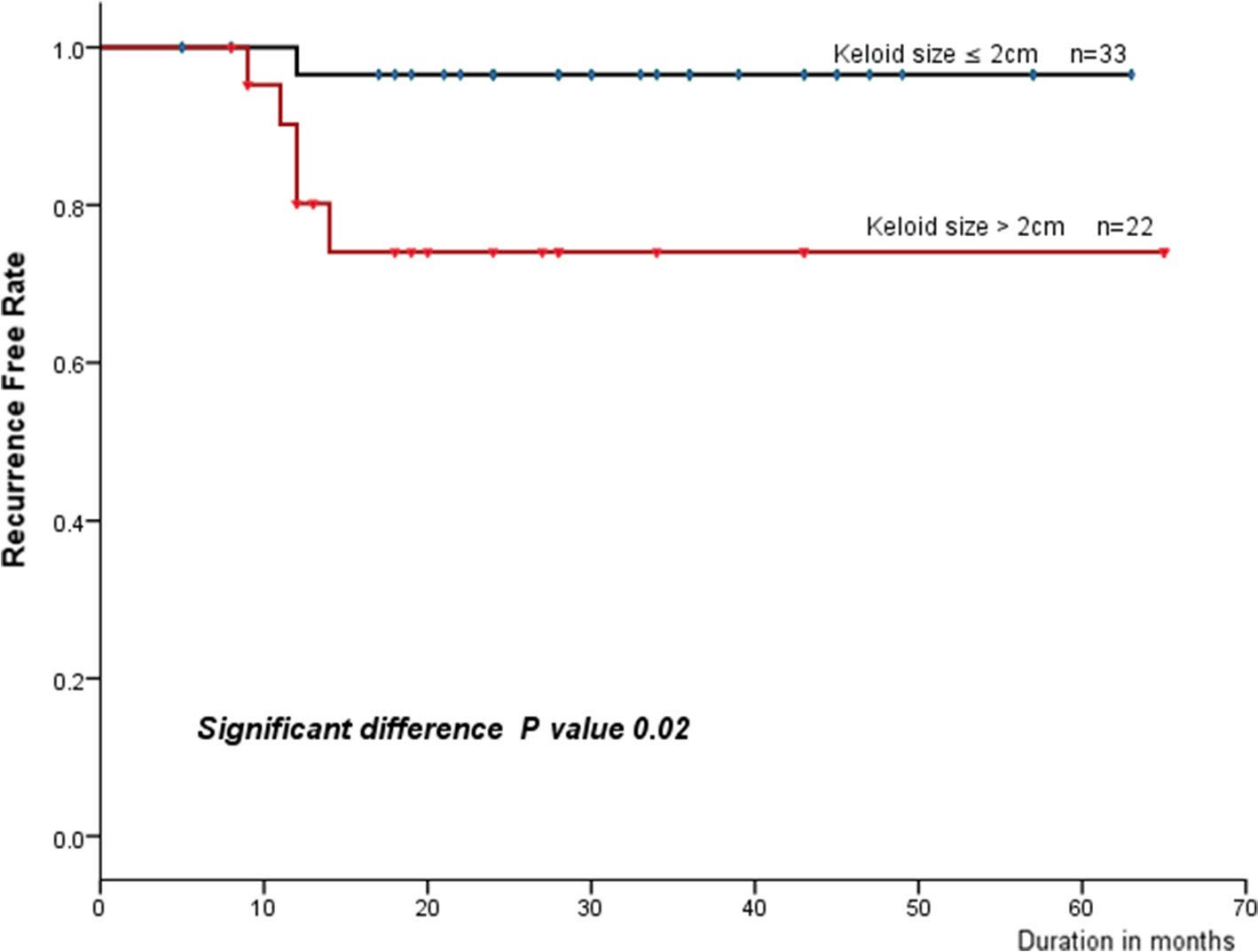
The effect of keloid size on the recurrence rate. The curve showed significant difference between keloid cases with size ≤ 2 cm and > 2 cm

EQD2 > 20 Gy did not show superiority compared to EQD2 ≤ 20 Gy. The 2y-RFR was 83 ± 8% for regimens with EQD2 ≤ 20 Gy and 92 ± 5% for regimens > 20 Gy with insignificant *p* value of 0.37. The cases that received orthovoltage showed 2y-RFR of 92 ± 4% compared to 72 ± 14% for cases that received electron beam, with a borderline insignificant difference (*p* value = 0.09). The size of the keloid of > 2 cm at the date of the last excision showed lower 2y-RFR of about 74 ± 10% compared to 97 ± 3% in cases of keloid ≤ 2 cm size with *p* value 0.02 (Fig. 4).

**Fig. 5:**
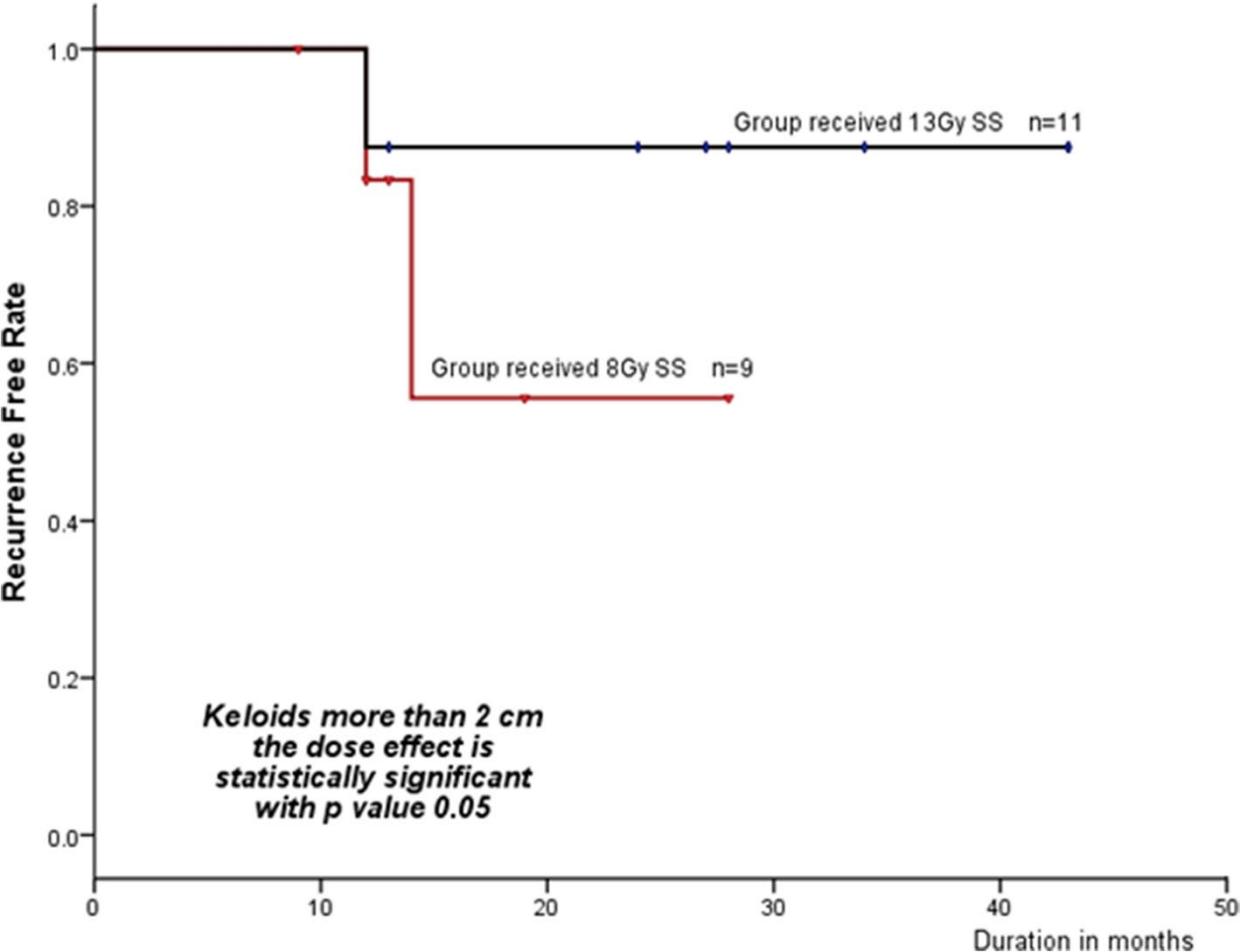
The effect of increasing the dose on recurrence for keloids > 2 cm. The curve showed significant effect of increasing dose to 13 Gy/1fx compared to 8 Gy/1fx on the recurrence free survival for keloid cases with size more than 2 cm

In a subset analysis for dose and keloid size, we targeted the keloid cases with size ≤ 2 cm and compared 8 Gy/1fx to 13 Gy/1fx with 2y-RFR 91 ± 9% versus 100 ± 0% (*p* value 0.3). Regarding keloid size more than 2 cm, the cases that received a dose of 13 Gy/1fx showed higher 2-year RFR (88 ± 12%) compared to the cases received 8 Gy/1fx (56 ± 24%) with a *p* value 0.05 as shown in Fig. 5.

**Fig. 6:**
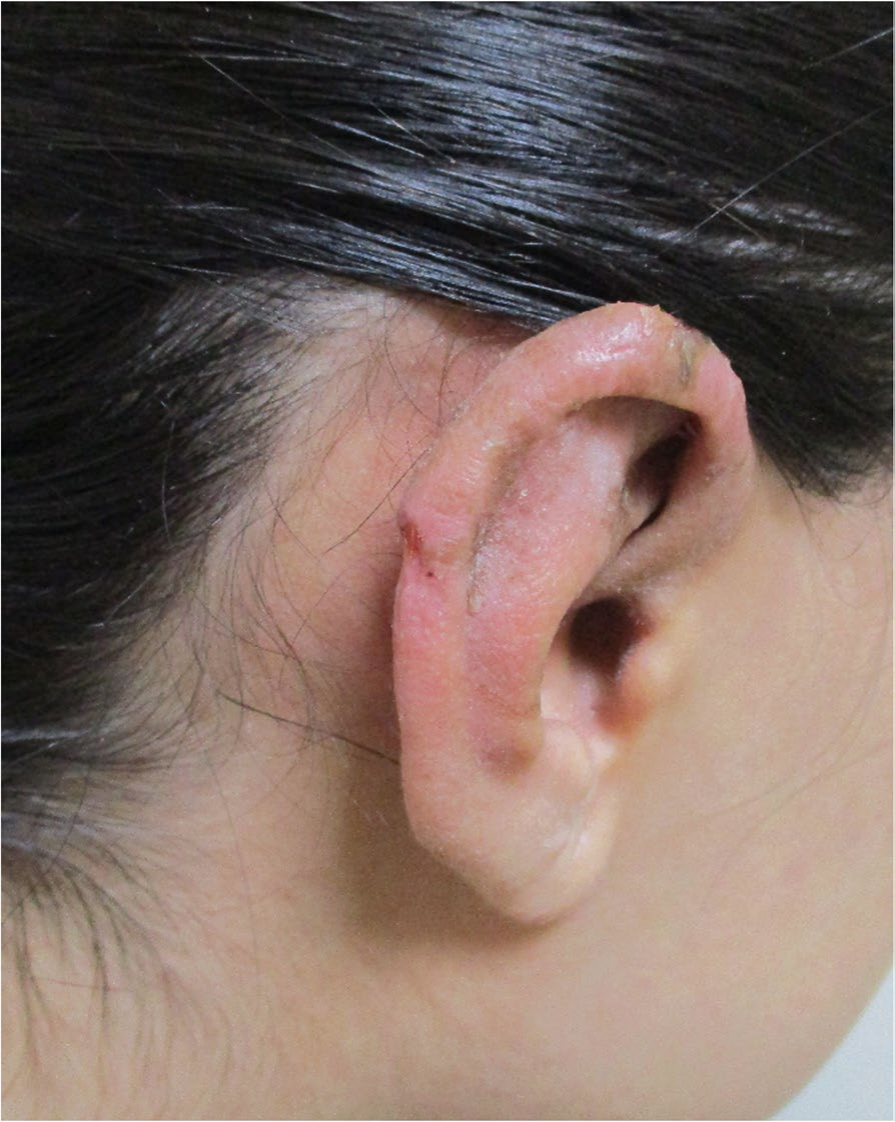
Acute radiation induced side effect. Patient with G1 radiation induced acute side effect as per RTOG grading scale

Sixty-seven percent of cases developed G1 acute skin reactions and only 9% of cases developed G2 acute reactions (Fig. 6). The G1 late skin reactions were reported in 14% only of cases. There are no G2–4 late skin reactions reported as shown in Table 2. The radiation-induced second malignancy is not reported in the studied cases with a mean follow-up of 35 months.

## Discussion

It is generally known that ear keloids do not regress spontaneously and are highly susceptible to recurrence following surgical excision. PORT is used since 1906 as an adjuvant to surgical excision and is considered a standard adjuvant approach with evidence of decreasing recurrence rate by more than 50% [2, 25, 26].

The weak point in most of the literature is the inclusion of a diversity of sites and even hypertrophic scars which are known to be more resistant to radiation. In our retrospective research, we studied only ear keloids to avoid the uncertainty of results and also being the commonest site affected worldwide. We aim to highlight the outcome of PORT and the effect of different prognostic factors like size and dose on the recurrence rate.

The treatment protocol at our department changed over the last 15 years. The following regimens were used; 8 Gy single shot (SS), 13 Gy SS, and many fractionated regimens as mentioned before, which make the comparison difficult however this is a common scenario in the literature [18, 20, 25, 26]. It is of significant importance to check the best dose regimen for ear keloids given the known lower recurrence rate for this site specifically compared to others.

We reported 2y-RFR of 88 ± 5% which is better compared to 79.4% recurrence-free published by Ragoowansi et al. who treated 35 ear lobe keloid by 10 Gy delivered by 100 kV orthovoltage within 24 h of surgery [27].

On the other side, our reported recurrence is higher compared to Ilias et al. who showed a 6% recurrence rate only for his 16 studied patients [29].

Wagner et al. again showed a higher recurrence rate of 21% compared to our results [30] and Kovalic also studied 113 keloids and showed a 27% recurrence rate. However, Kovalic’s study included 53% of the cases as hypertrophic scars and none ear sites which known to be more resistant as mentioned before [31].

The insignificant difference between low dose and high dose regimens used in our study is matching that of Wagner et al. who showed that the doses of 8–10 Gy are enough and comparable to higher doses [30]. Also, Kovalic et al. treated 75 keloids by 8 Gy only and proved its effectiveness for decreasing the recurrence rate by 50% [31]. Doornbos et al. showed a recurrence rate of around 10% and the dose less than 9 Gy is enough regardless of fractionation method [32]. In a retrospective study, Rei et al. compared 8 Gy/1fx, 15 Gy/3fx, and 10 Gy/2fx for ear lobe keloids with insignificant difference and recurrence rate of 9%, 14%, and 7%, respectively [33]. This evidence supporting the use of low to modest radiation doses in ear lobe keloid corresponds to our results.

The size of keloid is not well-studied in the literature as a prognostic factor; however, Kovalic et al. showed that keloids greater than 2 cm had a higher risk of recurrence. These results support our data that showed 2y-RFR 74 ± 10% for keloid cases of more than 2 cm size compared to 97 ± 3% for the group of keloids less than 2 cm with a *p* value of 0.02. Interestingly, increasing the delivered dose hides this difference in our study. The 2y-RFR for keloids of > 2 cm size received 13 Gy/1fx was 88 ± 12% compared to 56 ± 24% for the same group of keloids received 8 Gy/1fx with a significant *p* value of 0.05.

The timing of radiation in our study was not tested as all patients started the radiation within 24 h following surgery as per the recommendation of many studies [1, 13, 16].

As shown before, the orthovoltage showed marginally better 2y-RFR compared to electron beam; 92 ± 4% compared to 72 ± 14%. These results match Yang et al.’s data who showed the superiority of superficial x-ray therapy compared to electron beam therapy. They compared 14 patients who received PORT superficial intra-beam radiotherapy 8–10 Gy/2fx to 14 patients who received PORT by electron beam. There is no recurrence in the group that received superficial x-ray compared to 5 recurrences for the group of patients who received electron beam after a median follow-up of 22.5 months [34]. Also, Jones et al. showed a low recurrence rate of around 5% for patients who had surgical excision combined with platelet-rich plasma and postoperative superficial radiation therapy. But these data should be taken cautiously as the follow-up is only 3 months [35].

A meta-analysis of 72 studies by Mankaweski et al. including 9048 keloids reported the contrary. They showed no significant difference between superficial x-ray and electron beam with a 23% recurrence rate for both groups and a *p* value of 0.1 [18].

Seventy-six percent of our studied cases developed G1–2 acute side effects which are higher compared to 25% reported acute reactions by Wagner et al. [30]. However, late side effects reported in our study were 14% as G1 which is comparable to Sakamoto et al who treated 194 keloids with different dose regimens. They reported 19% late reaction in the form of hyperpigmentation, depigmentation, and telangiectasis with higher late adverse reactions up to 26% for patients who received doses of more than 20 Gy/5fx [36]. Also, in support of our results, Ragoowansi et al. showed no G3 acute or late reaction [27].

We did not report any second malignancy after a median follow-up of 35 months. This is similar to most of the studies; Mankaweski [18], Sakamoto [36], and Xu [37]. Ogawa et al. concluded no association between the 5 reported malignancies and the used radiotherapy for their studied keloid cases [23]. Berman and Nestor studied 96 keloids treated by superficial x-ray with a 10% recurrence rate and they did not report any second malignancy [38]. Also, Rishi et al. studied 40 keloids treated by electron beam and they did not report G3 side effects or second malignancies [39].

Another concept of the safety of PORT is the possibility of salvage of recurrent keloids following radiation by various treatment approaches. Assuring report by Rishi et al. supported the use of laser therapy and steroid injections for recurrent keloids following surgery and PORT [39].

## Conclusions

Surgical excision followed by PORT is an effective approach for the treatment of recurrent ear keloids. Low and modest doses of radiation are effective; however, keloids > 2 cm need higher doses of radiation to decrease the recurrence rate. There are no reported cases of second malignancy in our study supported by a long follow-up period. Although the safety of PORT is well documented, the optimization of radiation technique and dose should be of a major concern to avoid serious side effects. There are some limitations; lack of a control group and unavailability of side effects data for some cases that may underestimate PORT complications.

We recommend a prospective larger study to optimize radiation dose as a function of keloid size.

## Data Availability

Research data are stored in our institutional repository and will be shared upon request to the corresponding author.

## Abbreviations

RFR: Recurrence free rate
PORT: Postoperative radiotherapy
RT: Radiotherapy
Gy: Gray
Fx: Fraction
IRB: Institutional Review Board
kVp: Kilovoltage peak
kV: Kilovolt
MeV: Mega-electron volt
RTOG: Radiation Therapy Oncology Group
SPSS: Statistical package for social science
2y-RFR: 2 years recurrence free rate
EQ D2: Equivalent dose in 2-Gy fractions
BED: Biological effective dose
HVL: Half value layer
SS: Single shot
